# EMOTICOM: A Neuropsychological Test Battery to Evaluate Emotion, Motivation, Impulsivity, and Social Cognition

**DOI:** 10.3389/fnbeh.2016.00025

**Published:** 2016-02-24

**Authors:** Amy R. Bland, Jonathan P. Roiser, Mitul A. Mehta, Thea Schei, Heather Boland, Daniel K. Campbell-Meiklejohn, Richard A. Emsley, Marcus R. Munafo, Ian S. Penton-Voak, Ana Seara-Cardoso, Essi Viding, Valerie Voon, Barbara J. Sahakian, Trevor W. Robbins, Rebecca Elliott

**Affiliations:** ^1^Neuroscience and Psychiatry Unit, University of ManchesterManchester, UK; ^2^Institute of Cognitive Neuroscience, University College LondonLondon, UK; ^3^Institute of Psychiatry, Psychology and Neuroscience, Kings College LondonLondon, UK; ^4^Department of Psychology, University of CambridgeCambridge, UK; ^5^School of Psychology, University of SussexBrighton, UK; ^6^Institute of Population Health, University of ManchesterManchester, UK; ^7^School of Experimental Psychology, University of BristolBristol, UK; ^8^Psychology and Language Sciences, University College LondonLondon, UK; ^9^School of Psychology, University of MinhoGuimaraes, Portugal; ^10^Department of Psychiatry, University of CambridgeCambridge, UK; ^11^Behavioural and Clinical Neuroscience Institute, University of CambridgeCambridge, UK

**Keywords:** EMOTICOM, neuropsychological tests, social cognition, motivation and emotion, implusivity, neuropsychiatry, mental health

## Abstract

In mental health practice, both pharmacological and non-pharmacological treatments are aimed at improving neuropsychological symptoms, including cognitive and emotional impairments. However, at present there is no established neuropsychological test battery that comprehensively covers multiple affective domains relevant in a range of disorders. Our objective was to generate a standardized test battery, comprised of existing, adapted and novel tasks, to assess four core domains of affective cognition (emotion processing, motivation, impulsivity and social cognition) in order to facilitate and enhance treatment development and evaluation in a broad range of neuropsychiatric disorders. The battery was administered to 200 participants aged 18–50 years (50% female), 42 of whom were retested in order to assess reliability. An exploratory factor analysis identified 11 factors with eigenvalues greater than 1, which accounted for over 70% of the variance. Tasks showed moderate to excellent test-retest reliability and were not strongly correlated with demographic factors such as age or IQ. The EMOTICOM test battery is therefore a promising tool for the assessment of affective cognitive function in a range of contexts.

## Introduction

Mental health problems represent an extremely significant health burden, with global costs estimated at $2.5 trillion, projected to increase to $6.5 trillion by 2030, more than any other form of disease (Bloom et al., 2012; Fineberg et al., 2013). Impairments of emotional, motivational and social function are increasingly thought to be fundamental to the neurobehavioral pathology of psychiatric disorders and are becoming important targets for therapeutic intervention (Roiser et al., 2012). Major advances in treatment development will therefore be facilitated by well-designed, carefully validated measures of a comprehensive range of emotional, motivational, and social functions. This is critically important in clinical trials, of both of pharmacological and psychological interventions, which specifically aim to target emotional, motivational, and social processes. Currently, the outcome measures used in trials of such interventions are typically changes in clinical symptoms, and there is a pressing need for new outcome measures that quantitatively measure the effects of these treatments. A validated affective battery would also have important implications in other research contexts; for example, investigating cognitive profiles relevant to the NIMH Research Domain Criteria (RDoC) initiative that aims to create a new framework for mental health research (Insel et al., 2010; Sanislow et al., 2010) focusing on dimensions that cut across DSM diagnostic categories, investigating endophenotypes for genetic studies' or identifying biomarkers for high-risk individuals. However, at present there is no established neuropsychological test battery that offers a comprehensive assessment of “hot” cognitive functions.

Various individual tests have been developed and validated to test specific cognitive hypotheses. However, without standardization, it is difficult to make progress, replicate results, or identify gaps that need to be addressed (Elliott et al., 2011). Multi-center studies and clinical trials would benefit from a comprehensive, validated battery probing emotional, motivational, and social functions. The success of existing, standardized cognitive batteries highlights their recognized importance for assessing cognitive function. For example, the Cambridge Automated Neuropsychological Test Battery (CANTAB; Cambridge Cognition Ltd) has become a widely used battery in both academic research and clinical trials (Robbins et al., 1994, 1998; Cambridge Cognition Ltd). However, the focus is primarily on “cold” cognitive functions (Roiser and Sahakian, 2013) such as executive function, visuospatial memory and various types of attention. Here we generate normative data for a battery of neuropsychological tasks, which assesses a comprehensive range of processes relevant to affective cognition.

### Affective cognition

Affective cognition is a term used to describe aspects of cognitive function where stimuli have affective salience; the term “hot” cognition has been coined to distinguish these aspects of cognition from non-emotive “cold” cognitions (Roiser and Sahakian, 2013). Affective cognition, can be defined as reflecting an interface at which emotional and cognitive processes are integrated to generate behavior (Elliott et al., 2011).

Disrupted affective cognition is a core feature of many mental health disorders and cuts across DSM diagnostic categories. For example, biases in processing emotional stimuli have been observed in depression (Surguladze et al., 2004), anxiety (Mogg and Bradley, 2002), schizophrenia (Pomarol-Clotet et al., 2010), substance abuse (Ersche and Sahakian, 2007), eating disorders (Lovell et al., 1997), ADHD (Seymour et al., 2015), and phobic anxiety (Watts et al., 1986). Reward learning and motivation have been shown to be impaired in schizophrenia (Murray et al., 2007; Waltz et al., 2010), Parkinson's Disease (Voon et al., 2010), substance abuse (Park et al., 2010), affective disorders (Murphy et al., 2003), and ADHD (Thomas et al., 2015). Impulsivity has been in described in substance abuse (Voon et al., 2014), eating disorders (Mobbs et al., 2011), and ADHD (Malloy-Diniz et al., 2007). Finally, social cognition impairments have been demonstrated in autism (Happé and Frith, 1996), depression (Zahn et al., 2015), and schizophrenia (Fett et al., 2011). Social, emotional, and motivational aspects of cognition are thought to be key predictors of functional outcomes. Therefore, novel interventions targeting affective cognition may be effective for improving functional outcomes, as well as reducing symptoms. Examples include cognitive bias modification in depression (Baert et al., 2010; Roiser et al., 2012), social cognition training in schizophrenia (Combs et al., 2007), or pharmacological agents to promote social function such as oxytocin (Feifel et al., 2012).

A number of studies have explored the potential factor structure of affective cognition in mental health disorders. For example, the MATRICS project identified five sub-processes relevant to schizophrenia including, theory of mind, social perception, social knowledge, attributional bias, and emotional processing (Green et al., 2008). Others have identified four factors including perceiving emotions, facilitating thought, understanding emotions, and managing emotions (Mayer and Salovey, 1997; Mayer et al., 2003); or two factors including an emotional perception and understanding factor and an emotional facilitation and management factor (Eack et al., 2010).

However, despite a clear consensus that “hot” cognitive function is a multidimensional construct with many underlying sub-processes, no comprehensive battery assessing affective function is currently available. There are a number of batteries that include a limited number of affective tasks in predominately “cold” cognitive test batteries (e.g., CANTAB; www.cambridgecognition.com, MATRICS; www.matricsinc.org, CogState; www.cogstate.com, WebNeuro; www.brainresource.com). A recently developed explicit hot cognition battery, the Emotional Test Battery (ETB; www.p1vital.com), focuses on emotion processing tasks of particular relevance to depression.

In developing the test battery described here we chose to focus on four distinct domains of affective cognition: *emotion processing*, the ability to process and respond to affective stimuli, including emotional faces; *motivation*, the ability to learn, apply effort and make decisions driven by incentives; *impulsivity*, premature or risky responding; and *social cognition*, the ability to process information about situations involving interpersonal interactions. For each of these domains we piloted in 30 individuals a combination of novel, adapted and existing tasks designed to probe key underlying affective functions. We selected for inclusion in the final battery those tasks that were feasible in brief versions, readily understood and well-tolerated by participants and (for existing or adapted tasks) that elicited robust replication of previously observed effects. Further details of excluded tasks are available from the authors on request.

#### Emotional processing

##### Emotion recognition/categorization

Recognition of facial expressions is a widely-used paradigm in neuropsychiatry, particularly in studies of depressed patients who tend to rate ambiguous expressions as more negative (Bouhuys et al., 1999; Surguladze et al., 2004). Harmer et al. (2011) argues that emotional face recognition may be a sensitive biomarker for effective antidepressant treatment. We therefore aimed to develop a task that effectively probed emotional facial recognition. The Emotion Recognition Task (ERT) included in the CANTAB battery (Cambridge Cognition Ltd) has proven to be a promising task examining emotion recognition in clinical populations. However, in order to include an ERT in EMOTICOM with limited time available, we opted to focus on basic emotions; happy, sad, anger and fear and chose to exclude more complex emotions such as surprise and disgust. We also adapted the task to include two versions; one that assessed facial recognition, similarly to the original CANTAB ERT and one that more specifically assessed eye recognition. Including emotional eyes recognition was motivated by evidence supporting the “reading the mind from the eyes” test (Baron-Cohen et al., 2001) as an effective assessment of the ability to recognize the emotional state of others using just the expressions around the eyes. We further adapted the task to include control conditions, i.e., identifying the age of a face and eyes, to provide baseline measurement in neuroimaging investigations.

##### Attentional bias

Biased emotional attention can be effectively measured using the affective go/no-go test (Cambridge Cognition Ltd). Attentional biases have been observed in depression (Murphy et al., 1999; Erickson et al., 2005), mania (Murphy et al., 1999), anxiety disorders (Watts et al., 1986; Mogg et al., 1995), substance abuse (Ersche and Sahakian, 2007), and eating disorders (Lovell et al., 1997). Negative biases in processing emotional stimuli have been suggested as an important biomarker for antidepressant efficacy and may predict responses to both psychological and pharmacological interventions (Harmer et al., 2009; Roiser et al., 2012). We therefore adapted two versions of the Affective Go No-Go task: one similar to the CANTAB with word stimuli and one with face stimuli. The motivation for adapting the AGN to include faces was to potentially improve any cross-cultural, educational, and age influences on the word version. For example, emotionally salient word stimuli may require a minimum reading level that may not be suitable for use in children. Indeed, a facial version of the AGN has shown to be a promising tool in pediatric anxiety and depression (Ladouceur et al., 2006). Additionally, an emotionally cued Posner task (Posner, 1980) using eye gaze in emotional facial expressions was piloted as part of the development of the EMOTICOM battery but did not show significant condition effects.

##### Emotional memory

Biased emotional memory for personal experiences has been suggested as an important trait marker for depression (Brittlebank et al., 1993). Depressed patients also show a more general bias toward remembering negative information (Hamilton and Gotlib, 2008) and patients with schizophrenia show deficits in remembering positive stimuli (Herbener et al., 2008) suggesting a possible double dissociation between the two disorders. We therefore developed an emotional memory task that required an encoding phase presented at the start of the EMOTICOM battery and a retrieval phase presented at the end in order to assess biases in emotional memory. We also piloted an emotional working memory task using a spatial n-back (for review see Owen et al., 2005) with emotional faces, however this did not produce sufficient significant condition effects.

#### Motivation and reward

##### Reinforcement learning

Behavioral tests assessing reinforcement learning (RL) in humans are directly comparable to operant conditioning tasks used in animals (Roberts et al., 1988; Birrell and Brown, 2000). Human reinforcement learning tests typically involve learning which abstract stimuli predict winning or losing points or money (Owen et al., 1991; Pessiglione et al., 2006). Reinforcement learning, and corresponding responses in the brain's reward system, are reliably disrupted in several neuropsychiatric diseases, including schizophrenia (Murray et al., 2007; Waltz et al., 2010), Parkinson's Disease (Voon et al., 2010), alcohol dependence (Park et al., 2010), and depression (Murphy et al., 2003). One weakness of several tests is the conflation of reward and punishment learning (Cools et al., 2002). This is important, since reward and punishment may be subserved by separable, opponent processes in the brain (Daw et al., 2006). We therefore aimed to develop a novel reinforcement learning task that separated reward and punishment feedback in order to assess sensitivity to these independently.

##### Incentive motivation

Tests of incentive motivation measure how much effort an individual is prepared to exert to gain reward. The monetary incentive delay (MID) functional neuroimaging task features a speeded response to obtain a reward or avoid a loss (Knutson et al., 2001). However, the behavioral measure arising from this paradigm has seldom been shown to be altered by diagnosis or pharmacological manipulation (Knutson et al., 2004; Scheres et al., 2007). Indeed, the MID continually updates the threshold for success, which might reduce behavioral differences between conditions. Hence we aimed to develop an incentive motivation task that produced reliable behavioral differences that have the potential to provide important biomarkers for assessment and treatment interventions. We adapted the Salience Attribution Task (Roiser et al., 2009a) which has previously shown robust behavioral markers of adaptive motivational salience in Schizophrenia and developed a version that specifically evaluated motivation relating to reward and punishment separately.

##### Value-based choice

Tests of value-based choice investigate how subjects use different types of information (e.g., probability, reward, punishment) in order to guide economic decision-making. In contrast to tests of reinforcement learning, there is typically no learning component in tests of value-based choice. As such, the widely-used Iowa Gambling Task (Bechara et al., 1994) is not a specific test of value-based choice, since it also involves learning. The Cambridge Gamble Task (CGT: Rogers et al., 1999), part of the CANTAB suite of tests, asks subjects to decide on which of two options to bet, and to stake a certain percentage of their points on this bet. The CGT is sensitive to unipolar (Murphy, et al., 2001) and bipolar depression (Roiser et al., 2009b), schizophrenia (Hutton et al., 2002), and psychopharmacological manipulation (Rogers et al., 1999). However, it cannot determine whether decision-making is influenced by reward seeking or punishment avoidance. A later development (Rogers et al., 2003) can distinguish between these and is sensitive to several neuropsychiatric conditions (Roiser et al., 2006; Chandler et al., 2009) and psychopharmacological manipulations (Scarna et al., 2005), but includes a very restricted set of probabilities. We therefore adapted the CANTAB CGT (Cambridge Cognition Ltd) to investigate reward seeking and punishment avoidance separately.

#### Impulsivity

##### Waiting impulsivity

Coordination between initiation and inhibition of actions is required for successful behavior. Patients with ADHD (Aron and Poldrack, 2005), obsessive compulsive disorder (Malloy-Diniz et al., 2007), and schizophrenia (Kaladjian et al., 2007) show impairments in impulsivity. The four choice serial reaction time task (4-CSRTT) is a novel translation from the widely used 5-choice serial reaction time rodent task (5-CSRTT; Robbins, 2002). It has demonstrated clear deficits in substance abuse (Voon et al., 2014) and is sensitive to effects of dietary tryptophan depletion which is thought to reduce central 5-HT (Worbe et al., 2014). We therefore decided to incorporate the 4CSRTT (Voon et al., 2014) into the EMOTICOM battery which measures incentive motivation to rewards and premature responses elicited by anticipated reward.

##### Delay and probability discounting

Another aspect of impulsivity is the preference for immediate gratification, even when waiting longer might lead to higher absolute gain. Delay discounting is the progressive reduction in subjective value of a reinforcer with time. It can be assessed using two types of task—hypothetical or experiential. Hypothetical discounting tasks require choices between immediate (e.g., £1 now) and delayed (e.g., £5 in 1 month) rewards (Mazur, 1987; Green et al., 1996; Kirby, 2009). The experiential discounting tasks differs from hypothetical in that respondents directly experience the delay and receive the reward during the task (Reynolds and Schiffbauer, 2004). Patients with ADHD and substance use disorders show steeper discounting rates in such tasks, which also show good temporal stability similar to personality traits (Ohmura et al., 2006; Kirby, 2009). We therefore developed a computerized delay discounting task based on Richards et al.'s (1999) adjustment procedure.

#### Social cognition

##### Moral emotion

Moral emotions can be experimentally induced either in response to verbal descriptions or pictures of specific interpersonal behavior (Moll and de Oliveira-Souza, 2007) or behavior contravening normal social values (Zahn et al., 2009). Patients with ventromedial prefrontal (VMPFC) lesions show abnormal responses to hypothetical moral dilemmas (Ciaramelli et al., 2007; Koenigs et al., 2007) and patients with antisocial personality disorder (Blair, 1995) and Autism (Moran et al., 2011) show deficits in moral judgment. We developed a novel computerized Moral Emotions task that comprising of cartoon scenarios rather than lengthy vignettes that are more likely to be affected by reading ability, intelligence and age.

##### Theory of mind

Theory of Mind (TOM) refers to the ability to infer the mental states of others (Frith and Frith, 2003). A number of paradigms have been proposed to probe this function including false belief tasks (Frith and Corcoran, 1996), “faux pas” tests, visual jokes, understanding irony and the “Reading the Mind in the Eyes” test (Baron-Cohen et al., 2001; although note that this is most similar to an emotional recognition task—see above). Patients with autism typically show impaired TOM (Happé and Frith, 1996) and it is also sensitive to schizophrenia (Frith and Corcoran, 1996; Bora et al., 2009; Fett et al., 2011). While valuable in populations with overt impairment, existing TOM tasks are typically insensitive to variation in normal adult performance as most participants perform at ceiling. Therefore, we developed a complementary task that depicted ambiguous social situations with no right or wrong answer, thus allowing greater variation of responses in healthy volunteers. Rather than assessing whether participants have TOM ability, this task assesses the extent to which people choose to use TOM information.

##### Social economic exchange games

Economic games, such as the Ultimatum Game and Prisoners' Dilemma are popular tasks for exploring the neurobiology of social decision-making (King-Casas et al., 2005; Miller, 2005; Fehr and Camerer, 2007; Crockett, 2009). A number of patient groups have been studied using these games, including psychopathy (Koenigs et al., 2010; Rilling et al., 2015), schizophrenia (Agay et al., 2008), autism (Andari et al., 2010), depression (Pulcu et al., 2015), and borderline personality disorder (King-Casas et al., 2008; Seres et al., 2009; Unoka et al., 2009). It has been argued that these games may provide specific and sensitive biomarkers for social pathologies (Kishida et al., 2010). Traditionally, these games are long and involve a complex set-up with multiple players, which are unsuitable for neuropsychological testing. Therefore, we developed a simple one-player game of the Ultimatum Game and Prisoners' Dilemma, which probe social interaction within the context of a test battery.

##### Social decision making

Optimal decision-making in social contexts recruits a combination of associative and inferential computations. For example, one may have first-hand experience, one may observe choices of other people (or receive a recommendation), and one may infer the knowledge or intentions of the others to weight the influence of their decision. Therefore, we included the Inference Task which approximates the contribution of each of these processes to decision-making. Specifically, it employs the useful heuristic of confidence which can be used to infer the certainty of an agent's information and weight the influence of his/her endorsement on privately held beliefs (Thomas and McFadyen, 1995). The effects of such inferences on value computation are hypothesized to underlie the reassuring influence of another's confidence and generate distinct representations of value in the subject. Successful task performance requires cue combination, the integration of value computations, and theoretically, social inferences of other people's knowledge.

### Aims and objectives

The specific aims of the project were to: (a) generate a computerized test battery assessing multiple aspects of “hot” cognition; (b) demonstrate ease of administration, feasibility, and tolerability; (c) standardize the test battery in a large cohort of healthy volunteers, including an exploratory factor analysis to identify important, independent constructs; and (d) establish measurement stability in a smaller sample of healthy volunteers.

### Hypotheses

We hypothesized a factor analysis would reveal that the tasks would probe affective function best explained by a four factor model mapping onto *emotion processing, motivation, impulsivity*, and *social cognition*. We further hypothesized that tasks without a learning component would show at least moderate test-retest reliability.

## Materials and methods

### Participants

Two hundred healthy volunteers were assessed (see Table 1 for demographic characteristics), 42 of whom were re-tested within 5–10 days in order to assess test re-test reliability. This will furnish sufficient power to detect test-retest reliability of >0.35 (*p* = 0.05, 80% power). Potential participants were recruited via advertisements in the local community and on social media. Following telephone screening, participants were included if they met the following criteria: 18–50 years old; no self-reported previous or current psychiatric disorders, including depression, anxiety, eating disorders, and drug/alcohol dependence; no neurological disorders; no significant head injury resulting in unconsciousness; no current use of medication known to affect mood or cognition; no first-degree relatives suffering from any psychiatric disorders; smoked fewer than five cigarettes per day; drank less than the government guidelines for weekly alcohol intake (www.drinkaware.co.uk); and fluent in English. Participants completed the Brief Symptom Inventory (Derogatis and Melisaratos, 1983), meeting the criteria for adult non-patients across nine symptom dimensions; somatisation, obsessive compulsive, interpersonal sensitivity, depression, anxiety, hostility, phobic anxiety, paranoid ideation, and psychoticism. Participants were further interviewed using the Mini International Neuropsychiatric Interview (Sheehan et al., 1998) to exclude any psychopathology.

**Table 1:** **Demographic characteristics of sample (*N* = 200), stratified by age, IQ, gender, and ethnicity for the standardization of the EMOTICOM neuropsychological test battery**.

	**Mean**	**SD**
Age	26.66	9.81
Years in Education	14.40	2.01
WTAR IQ	112.18	6.29
**Gender**	**N**	**%**
Female	100	50
White	157	78.5
**ETHNICITY**		
Afro Caribbean	7	3.5
Asian-Indian	10	5
East-Asian	9	4.5
Mixed	9	4.5
Other	8	4

Eligible participants were invited to attend a 3.5-h appointment at the Neuroscience and Psychiatry Unit, University of Manchester or the Behavioral and Clinical Neuroscience Institute, University of Cambridge. Participants provided written informed consent after the study procedures were explained, and their IQ was estimated using the WTAR (Wechsler, 2008). This study was approved by the University of Manchester and the University of Cambridge Research Ethics Committees.

### Design

Participants completed 16 neuropsychological tests programmed in PsychoPy (Peirce, 2007) on a touchscreen laptop (Dell XT3). The tasks were administered in a quiet testing room over 3 h. Some participants chose to complete the tasks over two sessions no longer than 1 week apart. The tests were administered in a randomized sequence to eliminate systematic effects of fatigue. Participants were reimbursed for their time and travel expenses, they also received an additional bonus of up to £10, calculated on the basis of the average money won on tasks that involved a monetary incentive.

### Neuropsychological tasks

### Analysis

All analyses were performed with SPSS statistical software (IBM SPSS Statistics Version 20.0).

#### Factor analysis

The measures thought to be most reflective of the constructs investigated were standardized using *z*-scores (after transformation if appropriate) and entered into a factor analysis to determine the underlying latent variable structure of the data. Here, we conducted an exploratory factor analysis to identify the number of factors needed to maximize the amount of variance explained. An eigenvalue cut-off of 1 was used to determine whether a factor explained sufficient variability in the data. The method employed utilized varimax rotation with Kaiser normalization.

#### Reliability analysis

The reliability and stability of the tasks was assessed by comparing performance in 42 volunteers who competed the battery on two occasions, 5–10 days apart. Test–retest was assessed by calculating the average-measures intraclass correlation coefficient using a two-way mixed effects model, which controls for overall changes in performance between sessions (i.e., repetition effects). Different guidelines exist for the interpretation of the ICC. Here we take an ICC value of less than 0.40 to be poor, 0.41–0.59 as fair, 0.60–0.74 as good and values exceeding 0.75 as excellent (Fleis et al., 2003). These terms should be interpreted with caution as they do not take into account the confidence intervals of the ICC measure.

#### Correlation analysis

In an exploratory supplemental analysis, two tailed Pearson's correlations were used to correlate task performance with demographic measures such as age, IQ and years of education. Gender differences were examined using independent samples *t*-tests. The statistical significance of all correlations were corrected for multiple comparisons (*0.05/n; n* = *number of task variables*).

#### Task variables

For each task there are a number of possible outcome measures. For the factor analysis, test-retest analysis and correlations with demographic variables, which are the focus of the present publication, we chose the primary outcome measures outlined in Table 2.

**Table 2:** **Full list of neuropsychological tasks with outcome measures which are included in the EMOTICOM neuropsychological test battery**.

**EMOTION PROCESSING**	
Emotion recognition/categorization	**Task 1: Emotional Recognition Task (ERT)** We developed two versions of an ERT; one with full faces, and one with eyes only. In these tasks, the participant is shown a series of faces or eyes that appear on the screen briefly, and is asked to identify the emotion (happiness, sadness, anger or fear). In the control condition, participants are asked to identify the age of a face (child, young adult, middle aged, elderly).
	**Time to administer:** 12 min
	**Outcome Measures:** Accuracy scores were calculated for each facial emotion (happiness, sadness, anger, and fear). *Average accuracy* refers to average accuracy across all four emotions. *Affective bias* scores were calculated by subtracting accuracy for sad faces from accuracy from happy faces. This analysis was also performed for the eyes emotional recognition test.
	**Task 2: Emotional Intensity Morphing Task** This task assesses the point of emotional intensity at which participants can recognize a facial emotion. Participants view faces that either increase or decrease in emotional intensity and are instructed to respond when they either (a) detect the presence of emotion or (b) no longer detect the presence of emotion. The emotion that they were detecting was made explicit to participants. The task includes five different emotions: happiness, sadness, anger, fear, and disgust.
	**Time to administer:** 5 min
	**Outcome Measures:** The point of detection was calculated by taking the level of intensity in the facial expression needed in order to detect (increasing) or no longer detect (decreasing) each emotion. The *Average point of detection* refers to average point of detection across all five emotions. *Affective bias* scores were calculated by subtracting the point of detection for sad faces from point of detection from happy faces.
Attentional bias	**Task 3: Face Affective Go No-Go Task** This task assesses information processing biases for positive and negative facial expressions. The participant is told a target emotion (happy, sad, neutral), and asked to press a button only when the target emotion is present. The task consists of six blocks, each of which presents a series of faces: (1) happy target/sad distractor, (2) happy target/neutral distractor, (3) neutral target/happy distract, (4) neutral target/sad distract, (5) sad target/happy distract, and (6) sad target/neutral distract.
	**Time to administer:** 6 min
	**Outcome Measures:** Reaction times (RT) were calculated for all “hit” responses for each of the six conditions. *Affective bias* scores were calculated by subtracting the sad target/happy distract condition RT from the happy target/sad distractor condition RT.
	**Task 4: Word Affective Go No-Go** This task assesses information processing biases for positive, negative and neutral emotional words. Words were chosen based on their ratings in a pilot study in an independent cohort of 30 volunteers and were matched for valence, arousal, frequency and word length. Participants are given a target emotion (happy, sad, neutral), and asked to press a button only when the target emotion is present. Similarly to the faces affective go no go, the task consists of six blocks, each of which presents a series of words: (1) happy target/sad distractor, (2) happy target/neutral distractor, (3) neutral target/happy distractor, (4) neutral target/sad distractor, (5) sad target/happy distractor, and (6) sad target/neutral distractor. Note that this task is not the same as the Cambridge Cognition (www.cambridgecognition.com) word affective go no-go task.
	**Time to administer:** 6 min
	**Outcome Measures:** Reaction times (RT) were calculated for all “hit” responses for each of the six conditions. *Affective bias* scores were calculated by subtracting the sad target/happy distract condition RT from the happy target/sad distractor condition RT.
Emotional memory	**Task 5: The Emotional Memory Recognition Task** This task assesses biases in the recognition of emotional stimuli. During the *encoding* stage, participants are asked to rate images displaying positive, negative or neutral scenes, on valence and arousal intensity. Images were of scenes without people and were validated as positive, negative or neutral on the basis of pilot testing in an independent cohort of 30 volunteers. During the *retrieval* stage, images from the encoding phase are paired with new images. Participants are asked to indicate which image they saw previously. The encoding phase consists of 30 images (10 positive, 10 negative and 10 neutral) whilst the retrieval phase consists of 60 images (20 positive, 20 negative and 20 neutral), half of which were previously seen in the encoding phase.
	**Time to administer:** 5 min
	**Outcome Measures:** Valence and intensity ratings from the encoding phase were calculated for each valence condition; positive, negative and neutral. Retrieval affective bias was calculated by subtracting accuracy for negative stimuli from accuracy for positive stimuli.
**MOTIVATION AND REWARD**	
Reinforcement learning	**Task 6: Reinforcement Learning Task** This task separately assesses reward and punishment learning. Participants are shown colored circles, and asked to make a choice between the two based on which one they thought was more likely to win money and not lose money. Participants receive feedback and are continually updated on their total score. There are two conditions; one condition is a *no lose* condition whereby participants either win (£0.50 presented as 50p) or fail to win (0p). The second condition is a *no win* in condition whereby they lose (50p) or avoid losing (0p). Participants must learn, through sampling the circles, which of the two is the better option, with probabilities (unknown to participants) set at 70%/30%. In the *transfer* phase, all possible pairs of circles are presented and participants choose their preferred option. In this phase, no feedback is given.
	**Time to administer:** 12 min
	**Outcome Measures:** A reinforcement learning model was applied to the data. Learning rate (alpha) refers to how fast the participant learns new information. A high learning rate indicates that the participant incorporates new information more quickly. Alpha was calculated for win and loss conditions separately.
Incentive motivation	**Task 7: The Monetary Incentive Reward (MIR) Task** This task assesses effort to avoid punishments and gain rewards. Participants see a pair of identical circles displayed on the screen, shortly followed by a black box. Participants are instructed to make a response as soon as the black box appears. The circles contain colored lines, which indicate that on that trial they will either gain or lose money. The distance between the lines indicates the size of loss/gain. The faster they respond the more money they win or the less money they lose, and this relationship remains constant throughout the task.
	**Time to administer:** 10 min
	**Outcome Measures:** Reaction times were calculated for each condition; high win, low win, low loss and high loss. These reaction times were further standardized by subtracting each of the four conditions from the neutral “baseline” reaction time. High and low win reaction times were combined to produce the *average reaction time for wins*. Likewise high and low loss reaction times were combined to produce the *average reaction time for loss.*
	**Task 8: The Progressive Ratio Task** Progressive ratio tasks have been widely used to examine motivation in non-human subjects (Hodos, 1961; Bradshaw and Killeen, 2012). More recently, progressive ratio tasks have been adapted for use in humans using a variety of rewards (e.g., money, stimulants, food) to assess self-control and identify participants' motivational “breakpoint,” i.e., the maximum effort that a participant will expend in order to receive a reward (Roane, 2008). In this task participants are presented with four red squares on the screen and are instructed to select the square that differs in size to the other three. Participants are paid progressively less per trial as they continue with the task. They are also told that they can stop their participation in the task at any point, but that they still have to sit facing the screen for the remaining time (20 min minus the time they performed the task).
	**Time to administer:** 20 min
	**Outcome Measures:** The progressive Ratio task was adapted part way through the study therefore data is only presented for the adapted task (participant *n = 78*). The total number of trials was calculated in order to estimate the *breakpoint*, i.e., the point at which participants did not wish to continue with the task. *Running rate* was calculated as the time taken to complete the block of trials. The *post reinforcement pause* was the average time taken to initiate the next trial following a reward. Approximately 57% of participants completed the task therefore only allowing us to calculate a breakpoint for the remaining participants. Consequently, the progressive ratio task was not included in the factor analysis and test-retest reliability determinations.
Value-based choice	**Task 9: The adapted Cambridge Gambling Task** This task was developed to assess decision-making and risk-taking behavior, with reward and loss trials administered separately. On each trial, the participant is presented with a roulette wheel; a proportion of which is colored purple and a proportion of which is orange. There are 5 different proportions ranging from very certain to very uncertain. Participants must place a bet on the outcome they expect. A spinning pointer is then displayed, which lands on one of the colors, providing feedback for the participant. There are two conditions; a loss condition and a win condition which allows the separation of reward and punishment.
	**Time to administer:** 10 min
	**Outcome Measures:** The average value of chips placed on each level of probability was calculated separately for the win and loss conditions. Only choices of the most likely outcome were included. This was used to compute a *risk adjustment* (RA) score using the formula: Risk adjustment = (2^*^bet at 90%) + (1^*^bet at 80%) + (0^*^bet at 70%) - (1^*^bet at 60%) - (2^*^bet at 50%)/Average bet. RA was calculated for win and loss conditions separately.
**IMPULSIVITY**	
Waiting impulsivity	**Task 10: The four-choice serial reaction time task** This task (Voon et al., 2014) assesses visual attention, and ability to monitor and respond to unpredictable targets. Participants have to indicate a box, from 4 choices, in which a target has appeared.
	**Time to administer:** 25 min
	**Outcome Measures:** Data from 175 participants was utilized in the analyses due to initial technical problems. The motivational index was calculated by using the following formula: *motivational index = (baseline reaction time—post baseline reaction time)/ baseline reaction time.* The number of premature events was calculated as the combination of the number of premature releases (releasing the spacebar prematurely) and the number of premature responses (releasing the space bar prematurely and touching the screen).
Delay and probability discounting	**Task 11: The Discounting Task** This task assesses the rate of discounting across delays and probabilities. There are ten conditions; five levels of delay (0, 30, 90, 180, 365 days) and five levels of probability (100, 90, 75, 50, 25%). Participants must decide whether they would prefer a standard fixed amount (always £20) associated with a particular delay or probability, compared to an alternative amount definitely available immediately.
	**Time to administer:** 7 min
	**Outcome Measures:** Indifference points were calculated for each length of delay or degree of uncertainty. These indifference points refer to the amount of immediately available money that the participant considered to be equivalent to the delayed or uncertain reward. For delay discounting, the area under the curve was used to calculate the level of discounting using the following formula: Area under the curve = [(2-0)^*^((indifference point at 0 days + indifference point at 2 days)/2)]+[(30-2)^*^((indifference point at 2 days + indifference point at 30 days)/2)+[(180-30)^*^((indifference point at 3 days + indifference point at 180 days)/2)]+ [(365 -180)^*^((indifference point at 180 days+ indifference point at 365)/2)]. A smaller AUC, indicates more severe discounting of the delayed reward and thus greater impulsivity. A similar analysis was conducted for probability discounting, whereby smaller AUC indicates greater risk aversion.
**SOCIAL COGNITION**	
Moral emotion	**Task 12: The Moral Emotions task** This task uses cartoon figures to depict moral scenarios. Half of the scenarios depicted a deliberate harm whereas the remaining half depicted an accidental harm in order to explore the effect of intention upon moral emotions. Participants were asked to imagine how they would feel in the situation as either the actor or the victim, and rated the following emotions; guilt, shame, anger and feeling “bad.”
	**Time to administer:** 13 min
	**Outcome Measures:** The average rating for feeling bad was calculated across all conditions: victim vs. agent and intentional vs. unintentional. Agent ratings for guilt were also calculated.
Theory of Mind	**Task 13: Social Information Preference Test** This task assesses information sampling in socially ambiguous situations. Participants are shown a scene, with three faces (feelings), three thoughts and three facts about the scene hidden from view. Participants are able to select only four out of nine pieces of information to help resolve ambiguity. They then choose between three possible outcomes of the situation (negative, positive or neutral), which provides a measure of interpretational bias.
	**Time to administer:** 10 min
	**Outcome Measures:** The proportion of thoughts selected and the valence of the chosen outcome, positive, negative or neutral was calculated. The *affective bias* in interpretation was calculated by subtracting the proportion of negative outcomes chosen from the proportion of positive outcomes chosen.
Social economic exchange games	**Task 14: Prisoners' Dilemma** This task assesses cooperation with a computerized opponent. On each trial, participants must repeatedly press the space bar as fast as they can in order to fill a jar with coins. Each trial is manipulated so that the participant wins more coins, the opponent wins more coins, or they both win equal amounts. The coin totals are then combined and participants are instructed that they may either split or steal the total sum. Participants are told that if they both choose to split, they get half the money each, and if they both steal, they each get nothing. If they split and the opponent steals they get nothing and the opponent gets everything. Alternatively, if they steal and their opponent splits, they get everything and the opponent nothing. Participants are faced with three different opponents each with a different strategy: aggressive (tit for tat, but starts with steal), tit for two tats (starts with split, then changes behavior after the player has stolen two times consecutively) and a cooperative player who always splits.
	**Time to administer:** 10 min
	**Outcome Measures:** The average steal proportion was calculated as the proportion of trials that participants chose to steal from their opponent from the total number of trials across each type of opponent (aggressive, tit for two tats and cooperative).
	**Task 15: Ultimatum Game** This task assesses sensitivity to fairness and tendency to inflict punishment. Similarly to the Prisoner's Dilemma, participants initially complete a task in which they can win money. Here they can select 3 balls from a choice of 9 and depending on what colors are revealed behind the balls, participants can win money. Each trial is manipulated so that the participant wins more money, the opponent wins more money, or they both win equal amounts. This money is then combined with the opponent's total. Next, participants are informed whether they get to decide how the money is split or whether it is up to the opponent. If the opponent divides the money, the participant gets the choice to either accept or reject their offer. These offers have seven levels ranging from fair (50:50) to increasingly unfair (10:90). If the participant accepts, they each get the allotted amount, and if they reject, they both get nothing. When the participant divides, they can choose from four divisions differing in fairness (50:50, 40:60, 30:70, 20:80, and 10:90).
	**Time to administer:** 12 min
	**Outcome Measures:** The proportion of offers accepted was calculated as the number of trials that participants chose to accept the offer from their opponent from the total number of trials. Risk adjustment was further calculated by using the following formula: Risk adjustment = (2^*^acceptance at 50% offer) + (1^*^ acceptance at 40% offer) + (0^*^ acceptance at 30% offer) − (1^*^ acceptance at 20% offer) − (2^*^ acceptance at 10% offer)/Average offer. The average offer proposed refers to proportion of times participants chose each of the four levels of offer available.
	**Task 16: Inference Task** Participants initially view a series of face pairs and are instructed to touch the more confident of each pair. This confirms that they are able to read confidence in faces. Participants are then asked to guess the contents of a series of buckets (mostly red or mostly blue jellybeans), based on a combination of information sources. On each trial, the subject and the honest computer (who never lies) each take a sample from the bucket. The participant is provided with: a sample of eight jellybeans, the *answer* of the honest computer (based on its own sample; it does not know the participant's) and the *confidence* of the honest computer in the answer it provided. This confidence is expressed with a human facial expression, either positive or skeptical. Each bucket is different from the rest, and independently numbered. The sample, answer of the computer and computer confidence are independently manipulated. Optimally, the subject will be able to increase the computer's influence when it expresses confidence and decrease its influence when it is apparently unsure of its decision. Information inferred from the choice and confidence of the computer must also be combined with information directly observed in one's own
	**Time to administer:** 16 min
	**Outcome Measures:** The proportion of red and blue buckets chosen was calculated for each level of probability (i.e., number of red jellies in participants hand 1/8, 2/8, 3/8, 4/8, 5/8, 6/8, 7/8) and for each condition of computer choice (red or blue) and confidence (confident and unconfident). Area under the curve was calculated for each. The effect of probability refers to the area under the curve collapsed across all computer choice and confidence conditions. The effect of computer choice was calculated by subtracting the AUC when the computer chose blue from AUC then the computer chose red.

## Results

### Standardization

A summary of the means and standard deviations can be found in Table 3.

**Table 3:** **Summary of the means and standard deviations**.

**Domain and task**	**Test score used**	**Mean**	**SD**
**EMOTION**			
Facial recognition	Face: affective bias	9.48	19.76
	Eyes: affective bias	5.03	26.33
Emotional intensity	Increasing affective bias	−16.21	15.65
	Decreasing affective bias	3.18	14.51
Face affective go/no-go	Affective bias RT (ms)	−30.27	66.93
Word affective go/no-go	Affective bias RT (ms)	−3.30	195.10
Emotional memory	Retrieval affective bias	−4.05	10.66
	Average retrieval accuracy	93.67	7.83
**REWARD/MOTIVATION**			
Reinforcement learning	Win Learning rate	0.23	0.33
	Loss Learning rate	0.27	0.34
Monetary incentive reward	Win—neutral RT (ms)	34.50	34.42
	Loss—neutral RT (ms)	28.60	33.44
Adapted Cambridge gambling	Win risk adjustment	1.61	1.34
	Loss risk adjustment	1.94	1.17
Progressive ratio^a^	Breakpoint	78.12	32.35
	Post reinforcement pause (seconds)	2.00	0.74
**IMPULSIVITY**			
4CSRTT^b^	Motivational Index	0.16	0.15
Delay discounting	Delay discounting	3308.95	1928.79
	Probability discounting	989.71	255.20
**SOCIAL COGNITION**			
Moral emotions	Agent guilt ratings	79.68	12.22
	Feeling bad ratings	22.98	9.16
Information preference	Thoughts chosen	54.10	14.86
	Affective bias in outcome	10.59	20.57
Prisoners' dilemma	Average steal	39.63	28.39
Ultimatum game	Risk adjustment	2.06	1.80
	Value of offers proposed	36.80	10.07
Inference task	Effect of probability	388.00	65.84
	Effect of computer choice	177.75	129.37

a *Only 78 participants were included in the analyses for the Progressive ratio task due to an update to the task part way through the study*.

b *Only 175 participants were included in the correlation analyses for the 4CSRTT due to technical failure*.

#### Factor analysis

**Figure 1:**
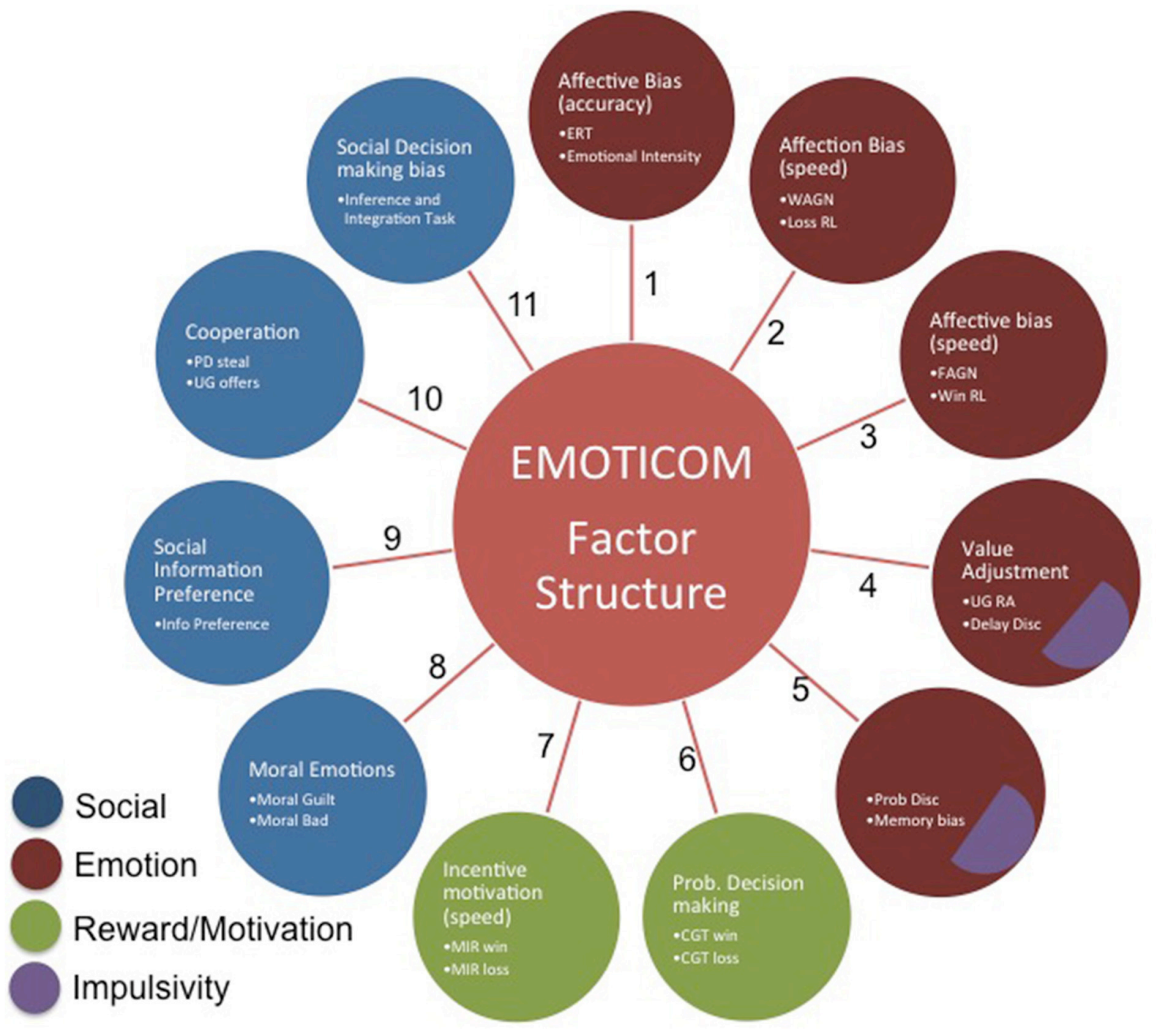
**Eleven-factor solution derived from an exploratory factor analysis**.

Data from all participants were entered into the factor analysis. The results of the varimax rotation for the tasks are shown in Table 4. An eleven-factor solution was derived based on eigenvalues greater than 1, which cumulatively accounted for 70% of the variance (see Figure 1). Only factor loadings greater than 0.40 are shown. Data were assessed for the adequacy of factor analytic methods. Bartlett's test was highly significant [χ${}_{\left(276\right)}^{2}=1071$.72, *p* < 0.001], suggesting that variable correlations did not form an identity matrix. Measures of sampling adequacy were also sufficient (KMO = 0.54).

**Table 4:** **Summary of the factor loadings for EMOTICOM tests on factors 1–11**.

**Test**	**Factors**										
	**1**	**2**	**3**	**4**	**5**	**6**	**7**	**8**	**9**	**10**	**11**
**EMOTIONAL RECOGNITION**											
Eyes affective bias	0.63										
Face affective bias	0.74										
**INTENSITY MORPHING**											
Increasing affective bias	−0.66										
Decreasing affective bias	0.62										
**WORDS AFFECTIVE GO/NO-GO**											
Affective bias (RT)		0.49									
**REINFORCEMENT LEARNING**											
Loss learning rate		−0.77									
Win learning rate			0.66								
**FACES AFFECTIVE GO/NO-GO**											
Affective bias (RT)			−0.74								
**ULTIMATUM GAME**											
Risk adjustment				0.78							
**DELAY DISCOUNTING**											
Delay discounting				−0.60							
Probability discounting					−0.49						
**EMOTIONAL MEMORY**											
Retrieval affective bias					0.75						
**CAMBRIDGE GAMBLING TASK**											
Win RA						0.79					
Loss RA						0.82					
**MONETARY INCENTIVE REWARD**											
Win-neutral RT							0.87				
Loss-neutral RT							0.83				
**MORAL EMOTIONS**											
Guilt rating (agent)								−0.87			
Feeling “bad” rating								0.89			
**INFORMATION PREFERENCE**											
Proportion thoughts									−0.70		
Outcome affective bias									0.65		
**PRISONERS DILEMMA**											
Steal rate (%)										−0.82	
**ULTIMATUM GAME**											
Value of offers proposed										0.83	
**INFERENCE TASK**											
Effect of probability											0.96
Effect of computer choice											0.95

Factor 1 represents affective biases in emotional recognition whereas Factors 2 and 3 capture affective biases in reaction times. Factor 4 contains tasks that have an element of value adjustment. Bias in emotional memory and probability discounting load onto Factor 5. Factor 6 represents measures of probabilistic decision making. Factor 7 represents latency measures of incentive motivation. Factor 8 represents social cognition, specifically moral emotions. Factor 9 represents social information preference and Factor 10 captures cooperation in social exchange games. Finally, Factor 11 loads onto social decision making. The 4CSRTT was omitted from the analysis in order to retain the full sample of participants; however, when running the factor analysis with the motivational index included, this variable loads onto factors 2 and 3 (affective biases in RTs).

#### Test-retest reliability

Test-retest reliability results are summarized in Table 5.

**Table 5:** **Test re-test reliability**.

**Domain and task (*N* = 200)**	**Test score used**	**Intraclass correlation coefficient**
**EMOTION**		
Facial recognition	Face: affective bias	0.86
	Eyes: affective bias	0.74
Emotional Intensity	Increasing affective bias	0.80
	Decreasing affective bias	0.73
Face affective go/no-go	Affective bias (RT)	0.34
Word affective go/no-go	Affective bias (RT)	0.44
Emotional memory	Retrieval affective bias	0.41
	Average retrieval accuracy	0.64
**REWARD/MOTIVATION**		
Reinforcement learning	Win learning rate	0.15
	Loss learning rate	−0.27
Monetary incentive reward	Win—neutral RT	0.37
	Loss—neutral RT	0.31
Adapted Cambridge gambling	Win risk adjustment	0.75
	Loss risk adjustment	0.75
**IMPULSIVITY**		
4CSRTT^a^	Motivational index	0.54
Delay discounting	Delay discounting	0.70
	Probability discounting	0.58
**SOCIAL COGNITION**		
Moral emotions	Agent guilt ratings	0.94
	Feeling bad ratings	0.87
Information preference	Proportion thoughts	0.62
	Affective bias in outcome	0.66
Prisoners' dilemma	Average steal rate	0.64
Ultimatum game	Risk adjustment	0.58
	Value of offers proposed	0.71
Inference task	Effect of probability	0.65
	Effect of computer choice	0.77

a *Only 32 participants were included into the reliability analyses for the 4CSRTT*.

#### Associations with demographic factors

Demographic factors associated with test performance are listed in Table 6.

**Table 6:** **Association between tasks and demographic characteristics**.

**Domain and task (*N* = 200)**	**Test score used**	**Age *(r)***	**IQ *(r)***	**Years in education *(r)***	**Gender *(t)***
**EMOTION**					
Facial recognition	Face: affective bias	0.27^*^	−0.16	−0.08	0.52
	Eyes: affective bias	0.37^*^	−0.09	−0.01	2.29
Emotional intensity	Increasing affective bias	−0.01	−0.01	−0.03	−1.30
	Decreasing affective bias	0.05	−0.05	−0.03	2.43
Face affective go/no-go	Affective bias (RT)	−0.01	−0.06	−0.09	−0.19
Word affective go/no-go	Affective bias (RT)	−0.11	−0.01	−0.01	1.23
Emotional memory	Retrieval affective bias	−0.01	0.04	0.04	−0.33
**REWARD/MOTIVATION**					
Reinforcement learning	Win learning rate	−0.02	0.02	0.13	−1.69
	Loss learning rate	−0.07	−0.04	0.03	−0.64
Monetary incentive reward	Win-neutral RT	0.01	−0.12	−0.04	−1.83
	Loss-neutral RT	0.02	−0.00	−0.04	−1.74
Cambridge gambling task	Win risk adjustment	−0.12	0.16	0.08	0.42
	Loss risk adjustment	−0.25^*^	0.27^*^	0.18	1.21
Progressive ratio^a^	Breakpoint	0.07	−0.07	−0.09	−0.35
	Post reinforcement pause	0.23	−0.32	−0.03	−0.67
**IMPULSIVITY**					
4 CSRTT^b^	Motivational index	−0.14	0.01	0.14	0.31
Delay discounting	Delay discounting	−0.16	0.27^*^	0.24^*^	1.28
	Probability discounting	0.03	−0.05	0.11	0.69
**SOCIAL COGNITION**					
Moral emotions	Agent guilt ratings	0.05	−0.02	−0.04	−4.02^*^^$^
	Feeling bad ratings	−0.12	0.11	0.14	1.96
Information preference	Proportion thoughts	−0.08	−0.05	−0.13	0.84
	Affective bias in outcome	0.07	0.01	0.07	−0.54
Prisoners dilemma	Average steal rate	−0.02	0.04	0.06	0.57
Ultimatum game	Risk adjustment	−0.13	0.13	0.09	−0.66
	Average value of offers proposed	0.19	−0.08	−0.09	0.22
Inference task	Effect of jelly probability	−0.16	0.15	0.18	−0.38
	Effect of computer choice	−0.19	0.15	0.18	−0.70

Results show Pearson correlations (r) or t-statistics (t) from independent t-test.

* *p < 0.002; N = 200*.

a *Only 78 participants were included in the correlation analyses for the Progressive ratio task due to an update to the task part way through the study*.

b *Only 175 participants were included in the correlation analyses for the 4CSRTT due to technical failure*.

$ *Females showed greater guilt ratings*.

## Discussion

Neuropsychological test batteries are vital tools for assessing the efficacy of treatment in neuropsychiatric disorders. In order to provide valid assessments of cognitive function, a neuropsychological test battery must possess good test retest reliability and examine a variety of cognitive functions with little redundancy. A further requirement of a test battery specifically assessing emotional and social function is that it should be (at least to some extent) independent of cognitive ability or IQ. In this paper we have presented data from 200 participants' performance to demonstrate that these requirements are met by the EMOTICOM neuropsychological test battery. This battery draws upon adaptations of pre-existing tasks as well as novel tasks in order to provide a comprehensive assessment of emotion processing, rewards and motivation, impulsivity and social cognition.

An exploratory factor analysis identified 11 factors, many of them loading onto a single task. Not all the factors are readily explicable and factors including variables with poor reliability should be viewed with considerable caution. We therefore do not attempt to draw conclusions about the meaning of individual factors. Rather we suggest that the central conclusion is simply that the tasks measure multiple constructs and therefore the battery has little redundancy. Our hypothesis of a four factor solution was categorially disproved suggesting that our prior operational concept of four domains was an over-simplification. This highlights the importance of administering multiple tests in order to assess these “hot” cognitive processes. Various reviews and meta-analyses have identified multiple domains of social cognition (Green and Leitman, 2008; Savla et al., 2012), however existing standardized batteries such as the MATRICS Consensus Cognitive Battery (MCCB; www.matricsinc.org) and CANTAB contain only one task targeting social cognition. The results presented here clearly indicate that there are different components of “hot” cognition that load onto multiple factors and therefore cannot be captured by a single test. Therefore, the EMOTICOM test battery provides a more comprehensive assessment of performance in a variety of affective processes and represents a significant advance over batteries including only a single test.

The majority of EMOTICOM tasks also showed moderate to excellent test-retest reliability. This is extremely important for assessing the efficacy of treatments and interventions, where it is important that differences in task performance can be attributed to effects of the interventions rather than methodological issues or random fluctuations. Furthermore, we demonstrate that our “hot” cognitive tasks have comparable retest reliability to traditional “cold” cognitive tasks (e.g., Lowe and Rabbitt, 1998). However, reliability of the reinforcement learning outcome variable was poor, consistent with previous observations that learning and memory tasks often do not exhibit good re-test reliability (Lowe and Rabbitt, 1998; Dikmen et al., 1999). Learning on these tasks transfers from the first session to the second. Such learning transfer results in significantly improved scores and lower variability at session 2, as we observed here. Given this poor reliability, the EMOTICOM reinforcement learning task could potentially be improved by creating parallel versions using different stimuli, although participants are still likely to be able to generalize rule-learning from the first session. Reliability of bias measures in the Affective Go No Go and Monetary Incentive Reward tasks were also poor. Bias reliability scores in reaction times are often reported to be much lower than mean RTs from each condition (Eide et al., 2002; Strauss et al., 2005; Brown et al., 2014) and our results are therefore comparable with previous studies. Poor test-retest reliability on specific tasks suggests caution in using these measures in longitudinal contexts with healthy volunteers, however it does not preclude the use of these tasks in between-group studies with patient populations.

The majority of EMOTICOM tasks were not strongly correlated with demographic factors such as age, years in education or IQ suggesting that performance of these tasks is not dependent upon general intellectual function. There are a few exceptions: the risk adjustment measure from the loss condition in the adapted Cambridge Gambling Task and the delay discounting measures were correlated with IQ, with delay discounting also being correlated with years in education. Previous studies have also suggested that gambling (Demaree et al., 2010; Webb et al., 2014) and delay discounting (Shamosh and Gray, 2008) correlate with intelligence. Therefore, it is recommended that studies using these measures take particular care to control for IQ and years of education. Interestingly we observed emotional bias measures in the face and eyes emotional recognition task to be significantly correlated with age, such that biases became more positive with increasing age. This finding supports a line of research that has recently gathered momentum, with many recent studies demonstrating that people attend to and remember positive information more as they get older (e.g., Mather and Carstensen, 2003; Reed and Carstensen, 2012). In spite of a prevailing view that hot cognitive tests are dependent on gender, we only observed a significant effect of gender in the Moral Emotions Task, whereby females show greater guilt ratings compared to males. This is in line with existing meta-analyses showing that women tend to experience negative emotions, such as guilt, more intensely than men (Else-Quest et al., 2012). This task may therefore be useful in understanding gender differences in treatment outcomes, particularly in terms of self-blame biases and their suggested link to a vulnerability to depression (Green et al., 2013).

### Limitations

The ethnic characteristics of our sample of 200 participants was representative of the UK demographic (Office for National Statistics, 2011). Nevertheless, caution is recommended in generalizing these findings across cultures. Evidence suggests that cultural variations are evident in affective cognition. For example cultural variations have been observed in emotional facial recognition (Prado et al., 2014) economical games such as the Ultimatum Game and Prisoners' Dilemma (Oosterbeek et al., 2004; Wong and Hong, 2005) and arguably moral judgment (Gibbs et al., 2007). Such differences observed in performance across cultures suggest care in generalizing performance on UK validated and standardized tasks to other cultures. Another limitation is that we were not able to enter all the task variables into the factor analysis due to the reduced number of participants who completed some of the tasks. For instance, the progressive ratio parameters were improved part way through the study and so data were only available from 78 participants. Similarly, only a subset of participants completed the 4CSRTT. Therefore, in order to increase power and retain the full participant sample, the decision was made to omit these measures from factor analysis. A limitation of the test-retest reliability component was that we only assessed reliability over a short duration; in future it will important to assess longer durations to determine the potential value of the tasks in different intervention contexts.

In summary, we have demonstrated the potential power of the EMOTICOM test battery for the assessment of affective cognitive function. We have shown that affective cognition is far from a unitary construct, implying that assessment of multiple aspects of affective cognition is required. Our 16 task battery has little redundancy from the 11 factor underlying structure. We have also demonstrated that the majority of tasks have moderate to excellent test-retest reliability and are not strongly correlated with demographic factors such as IQ. We therefore conclude that the EMOTICOM test battery meets certain key criteria for a useful and valid tool with potential utility in clinical trials and studies investigating psychiatric disorders and relevant treatment interventions.

Important future directions include validation in patients and validation in intervention studies in both healthy controls and patients in order to further investigate the utility of EMOTICOM test battery, and diagnosis-appropriate subsets of tasks, as an investigative tool in mental health research. This will enable us assess which tasks are most valid, sensitive and reliable for use in particular patient populations and which can be used as outcome measures in intervention trials.

## Funding

This work was supported by the MRC under Grant MR/J011894/1.

### Conflict of interest statement

The authors declare that the research was conducted in the absence of any commercial or financial relationships that could be construed as a potential conflict of interest. The reviewer YW and the handling Editor declared their shared affiliation, and the handling Editor states that the process nevertheless met the standards of a fair and objective review.
